# Bacterial and Fungal Gut Community Dynamics Over the First 5 Years of Life in Predominantly Rural Communities in Ghana

**DOI:** 10.3389/fmicb.2021.664407

**Published:** 2021-07-06

**Authors:** Nelly Amenyogbe, Dennis Adu-Gyasi, Yeetey Enuameh, Kwaku Poku Asante, Dennis Gyasi Konadu, Seyram Kaali, David Dosoo, Pinaki Panigrahi, Tobias R. Kollmann, William W. Mohn, Seth Owusu-Agyei

**Affiliations:** ^1^Department of Experimental Medicine, University of British Columbia, Vancouver, BC, Canada; ^2^Systems Vaccinology, Telethon Kids Institute, Perth, WA, Australia; ^3^Research and Development Division, Ghana Health Service, Kintampo Health Research Centre, Kintampo North, Ghana; ^4^Department of Epidemiology and Biostatistics, School of Public Health, Kwame Nkrumah University of Science and Technology, Kumasi, Ghana; ^5^Pediatrics Academic Department, Georgetown University Medical Centre, Washington, DC, United States; ^6^Department of Pediatrics, University of British Columbia, Vancouver, BC, Canada; ^7^Department of Microbiology and Immunology, Life Sciences Institute, University of British Columbia, Vancouver, BC, Canada; ^8^Institute of Health Research, University of Health and Allied Sciences, Ho, Ghana

**Keywords:** breast milk, child, fungi, bacteria, post partum microbiome

## Abstract

**Background:**

Bacterial and fungal microbiotas are increasingly recognized as important in health and disease starting early in life. However, microbiota composition has not yet been investigated in most rural, low-resource settings, and in such settings, bacterial and fungal microbiotas have not been compared. Thus, we applied 16S and ITS2 amplicon sequencing, respectively, to investigate bacterial and fungal fecal microbiotas in rural Ghanaian children cross-sectionally from birth to 5 years of age. Corresponding maternal fecal and breast milk microbiotas were additionally investigated.

**Results:**

While bacterial communities differed systematically across the age spectrum in composition and diversity, the same was not observed for the fungal microbiota. We also identified a novel and dramatic change in the maternal postpartum microbiota. This change included much higher abundance of *Escherichia coli* and much lower abundance of *Prevotella* in the first vs. fourth week postpartum. While infants shared more bacterial taxa with their mother’s stool and breast milk than with those of unrelated mothers, there were far fewer shared fungal taxa.

**Conclusion:**

Given the known ability of commensal fungi to influence host health, the distinct pattern of their acquisition likely has important health consequences. Similarly, the dynamics of mothers’ bacterial microbiotas around the time of birth may have important consequences for their children’s health. Both topics require further study.

## Introduction

The dynamics of the bacterial gut microbiota have been well studied but mostly in resource-rich settings. These surveys have shown microbial diversity to increase with age and reach adult-like profiles by 2–3 years (Palmer et al., 2007; Yatsunenko et al., 2012; Subramanian et al., 2014; Backhed et al., 2015; Bokulich et al., 2016; Stewart et al., 2018). The breast milk bacterial microbiota has also been studied using culture-based (Jimenez et al., 2008; Solis et al., 2010; Jost et al., 2013) and culture-independent (Cabrera-Rubio et al., 2012; Moossavi et al., 2019) approaches, which revealed its potential to seed the infant intestinal microbiota (Martin et al., 2012). These findings together with disease-specific surveys have identified the bacterial microbiota as a key factor shaping health in early life (Arrieta et al., 2014).

In contrast, the fungal gut microbiota has been far less studied, despite emerging evidence that fungi are also key modulators of health and disease (Jiang et al., 2017; Shao et al., 2019). Examples include recent mouse studies demonstrating that fungi effectively calibrate mucosal immune responses with an emphasis on Th17 immunity and neutrophil function (Shao et al., 2019) and that colonization with *Candida albicans* increases survival of systemic viral challenge (Jiang et al., 2017). Sequence-based surveys of infant fungal microbiotas have also revealed that a higher abundance of specific fungal taxa is associated with the development of allergic disease later in childhood (Arrieta et al., 2018). The human fungal microbiota in early life has been surveyed in very few studies and in select populations: Europe (Norway, Florence, and Luxembourg) and America (Puerto Rico and Ecuador) (Strati et al., 2016; Schei et al., 2017; Wampach et al., 2017; Arrieta et al., 2018; Ward et al., 2018). Based on diverse study designs, *Candida*, *Aspergillus*, and *Saccharomyces* feature prominently in newborns and older infants. There is, however, no clear age-dependent trajectory of fungal microbiota taxonomic composition (Strati et al., 2016; Schei et al., 2017; Wampach et al., 2017) or diversity (Strati et al., 2016; Wampach et al., 2017) in the first 2 years of life, aside from lower diversity in 10-day-old newborns vs. 3-month-olds or their mothers (Schei et al., 2017). There were also no clear trajectories identified during the first month of life in a more granular assessment of the newborn fungal microbiota (Ward et al., 2018).

The breast milk fungal microbiota has only recently been profiled using a culture-independent approach (Boix-Amoros et al., 2019), revealing dominance by *Malassezia* spp. also commonly found on skin and compositional differences across diverse populations, which were confirmed in separate studies (Heisel et al., 2019; Moossavi et al., 2020). Beyond this, the fungal composition of human breast milk has not been investigated.

Despite the importance of both the bacterial and fungal gut microbiota in influencing the developmental trajectory toward health or disease, to our knowledge, these two have never been jointly investigated in early life, which is likely the most important period of development. Equally surprising, despite the well-established fact that the maternal stool and breast milk microbiotas shape the newborn’s microbiota, no study so far has captured this interaction between mother and infant across the bacterial and fungal domains. We thus conducted a cross-sectional bacterial and fungal microbiota survey encompassing fecal microbiotas of children in the first 5 years of life plus fecal and breast milk microbiotas of mothers of children one month and younger.

## Materials and Methods

### Ethics Approval and Consent to Participate

All work in this study was in accordance with ethical guidelines at the University of British Columbia and done under the approved ethics protocol number H11-01423. Separate ethical approval was obtained from the Kintampo Health Research Centre Institutional Ethics Committee (KHRCIEC_2016-14).

### Study Participants

Study participants were recruited in the Kintampo North Municipality, located in the former Brong Ahafo Region (now Bono-East Region) in the middle-belt of Ghana, and serving as part of the Kintampo Health Research Centre (KHRC) study area. Prospective participants were selected from the database of the Kintampo Health and Demographic Surveillance System (KHDSS). Expectant mothers of newborn participants were approached during pregnancy, or mothers of older children, who were recruited into the study were approached to participate in the study. Inclusion criteria for this study were for participants to be (a) living in the study area, (b) healthy with no known congenital defects, (c) under 63 months, and (d) to have provided written informed consent. An exclusion criterion was refusal to provide informed consent. Study participant recruitment was stratified by the age groups outlined: the newborn period [day of life (DOL) 0–5; DOL 13–17; DOL 26–35]; 3 months (DOL 83–115), 6 months (DOL 165–200), 1 year; month of life (MOL) 11–13; 2 years, MOL 22–26; 3 years, MOL 33–39; and 5 years, MOL 57–63.

### Stool and Breast Milk Sample Collection

Field workers supplied mothers with sterile containers and scoops to collect stool samples from soiled diapers. Stool samples were collected from the diapers of children with sterile plastic spoons transported to the clinical laboratory at the KHRC. Additionally, mothers of participants who were 0–1 or 4–5 weeks old and were exclusively breastfed were asked to provide stool and breast milk samples in supplied sterile containers. All samples collected in the study were kept in cold boxes with ice packs and sent to the laboratory within 2 h. At the clinical laboratory, stool and breast milk samples were split into multiple 1.0-ml aliquots and stored at −80°C until further analysis. Biospecimens were transported to the University of British Columbia on dry ice with temperature monitoring *via* World Courier Inc.

### Stool DNA Extraction for Amplicon Sequencing

DNA was extracted from swabs using the MagAttract PowerSoil DNA KF kit (Qiagen Cat. No. 27000-4-KF) using the manufacturer’s protocol for the KingFisher Flex platform with the following modification using approximately 200 mg bulk stool loaded into each 96-well plate with sterile wooden picks.

### Breast Milk DNA Extraction for Amplicon Sequencing

Total DNA was extracted from 0.7–1.0 mL breast milk using the Qiagen DNease PowerSoil DNA extraction kit (Qiagen Cat. 12830-50) with the following modifications: frozen breast milk samples were thawed on ice and transferred to 2.0 ml screw-top tubes (VWR Cat. 211-0440) and spun for 10 min at 4°C at 20,000 g to pellet all particles. The lipid portion of the milk remained on top of the aqueous phase after centrifugation. The lipid layer was retained, and the aqueous phase between the pellet and whey layer was carefully removed with a 200-μL micropipette. The contents of one Qiagen 0.1-mm glass bead tube (Qiagen 13118-50) was then added to each sample with 500 μL Bead Solution and 200 μL phenol:chloroform:IAA pH 7–8 (Ambion Cat. AM 9730, pH adjusted with included Tris buffer). Then, 60 μL of C1 was added, and samples were homogenized in a FastPrep bead mill for 30 s at 5.5 m/s for two cycles with a 5-min wait in between. The remainder of the protocol was carried out according to manufacturer’s instructions, and samples were eluted in 2 × 30 μL (total of 60 μL).

### 16S Amplicon Sequencing

Samples were submitted to Microbiome Insights Inc. (Vancouver, BC, Canada) for PCR amplification and DNA sequencing targeting the V4 region of the 16S rRNA gene. Library preparation was done using a previously published protocol (Kozich et al., 2013) with the details and product information outlined below. Here, 10 μL of the final product was used to normalize to 1–2 ng/μL using the SepalPrep Normalization Prep Plate Kit (Thermo Fisher Cat. A1051001), and 5 μL of each normalized sample was pooled into a single library per 96-well plate. Library pools were further concentrated using the DNA Clean and Concentrator kit (Zymo Cat. D4013). A dilution series was performed for each of the pooled libraries for subsequent quality control (QC) steps. Each pool was analyzed using the Agilent Bioanalyzer using the High Sensitivity DS DNA assay (Agilent Cat. 5047-4626) to determine approximate fragment size and to verify library integrity. Library pools with unintended amplicons were purified using the Qiagen QIAquick Gel Extraction Kit (Qiagen Cat. 28706). Pooled library concentrations were determined using the KAPA Library Quantification Kit for Illumina (KAPA Cat. KK4824). The final libraries were loaded at 8 pM, with an additional PhiX spike-in of 20%. The amplicon library was sequenced on the MiSeq using the MiSeq 500 Cycle V2 Reagent Kit (Illumina Cat. MS-102-2003).

Paired-end reads were assembled using MOTHUR version 1.37.2, following the MiSeq SOP (Schloss et al., 2009; accessed Aug 2018)^1^. Operational taxonomic units (OTUs) were clustered at 97% identity and classified using the GreenGenes 13_8_99 database (DeSantis et al., 2006).

### ITS2 Amplicon Sequencing

Processing for ITS2 amplicon sequencing was identical to that used for 16S amplicon sequencing, except with the following differences: Primers and PCR conditions were previously described (Gweon et al., 2015). Paired-end reads were trimmed at the non-overlapping ends, and high-quality reads were classified using UNITE (v. 7.1) (Koljalg et al., 2005).

### 16S and ITS2 Sequencing Quality Control

The potential for contamination was addressed by co-sequencing DNA amplified from specimens and from four template-free PCR controls and extraction kit reagents processed the same way as the specimens (two for breast milk specimens). Two positive controls, consisting of cloned SUP05 DNA, were also included (number of copies = 2^∗^10^6). Breast milk samples were extracted and PCR amplified separately from the stool samples but sequenced on the same lane. Given the different distributions of contaminating OTUs from breast milk blanks compared to stool, QC was done for the breast milk samples separately. Contaminating OTUs can come from two main sources: from PCR and extraction reagents and carryover from neighboring samples during the extraction and amplification process. OTUs were considered reagent contaminants and removed from the dataset if they were present in over 50% of blank samples and their count geometric mean plus one standard deviation was greater than that in the samples. Using this approach, OTUs with high counts in many samples (mainly including taxa commonly known to be highly abundant in these sample types) were not flagged for removal, despite their presence in blanks. For OTUs whose mean counts were high in the samples but also found in over 50% of blanks, the mean count plus standard deviation from the blanks was subtracted from each of the samples. Samples with total counts under 1,000 were also removed. Once filtered, data from breast milk and stool were recombined for further analysis.

### Statistical Analysis

#### Alpha Diversity

We used the *phyloseq* R package (Charlop-Powers and Brady, 2015) to compute alpha and beta diversity. We estimated microbial diversity with abundance-dependent (Shannon) and independent (Observed richness) metrics after subsampling the OTU table to account for unequal sampling depth. To test for relationships between age, we used the Wilcoxon test comparing each age bin to the Mothers of one-month-old (DOL 26–35) infants, as this was considered the adult microbiota group. *p*-values were adjusted using the Bonferroni correction, and adjusted *p*-values under 0.05 were considered significant.

#### Community Composition

Beta diversity was computed using the Bray–Curtis index. Sample clustering was visualized using non-metric-multidimensional scaling (NMDS). To test whether child age (all age bins except maternal samples) explained community composition, we used the Adonis test from R package *vegan* (Oksanen et al., 2017) using age in days calculated between the time interval between participant’s date of birth and visit date. To test if community composition between 5-year-olds (age bin MOL 57–63; 5 years) and younger children differed, we computed the Bray–Curtis distance of individuals in each bin to their distance to all individuals in the MOL 57–63 bin. The Wilcoxon rank-sum test was then used to compare distances between each bin to the median distance among the 5-year-olds to others in the same bin. *p*-values were adjusted using the Bonferroni correction, and adjusted *p*-values under 0.05 were considered significant.

#### Differential Abundance

To identify OTUs differentially abundant between mothers of 0–5-day-old vs. 26–35-day-old infants, we used the Wald test implemented in the R package *DESeq2* (Love et al., 2014). Differential abundance was considered significant if the Benjamini–Hochberg adjusted *p*-value was less than 0.05. To further visualize select bacterial genera differentially abundant between maternal groups and their abundance across the age spectrum, we aggregated OTU counts by genus-level assignment and confirmed that the genera differed between groups using the Wilcoxon rank-sum test.

#### Shared Operational Taxonomic Units Between Mother and Infant

For shared OTU analysis, OTUs were considered present if their relative abundance was over 0.01%. For stool and breast milk separately, we calculated the number of shared OTUs between newborns and their mothers and newborns and unrelated mothers. Because the number of unrelated pairs was much greater than related pairs, we randomly subsampled the unrelated pairs, selecting the same number as related pairs in each analysis. The median number of shared OTUs between related and unrelated pairs and 0–5 and 25–36 days postpartum was then compared using the Wilcoxon rank-sum test.

## Results

### Sample Summary: Amplicon Sequencing

After quality filtering, we retained a total of 2.9 million reads, representing 4,492 bacterial OTUs. These OTUs represented 435 unique taxa classified at the genus level or above. Five samples yielded fewer than 1,000 reads and were excluded from downstream analysis. The remainder yielded between 2,600 and 31,200 reads per sample, with most fecal samples yielding greater than 10,000 (Supplementary Figure 1A).

After quality filtering, we retained a total of 2.5 million reads representing 1,772 fungal OTUs, representing 375 unique taxa classified at the genus level or above. Fifty-nine samples contained fewer than 1,000 reads and were excluded from downstream analysis. The remainder contained between 1,200 and 96,500 reads per sample (Supplementary Figure 1B). Samples with no detectable fungi over our count threshold were found in both infant and adult stool samples alike (Supplementary Figure 1C). The proportion of samples in each age stratification with no detectable fungi was highly variable but usually below 50%.

### Bacterial and Fungal Alpha Diversity Across the Age Spectrum

**FIGURE 1:**
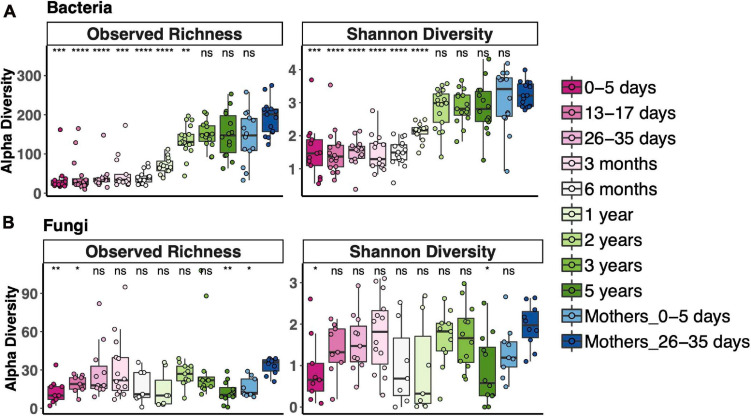
Alpha diversity across the age spectrum for bacterial fecal communities **(A)** and fungal fecal communities **(B)**. Coding indicates values significantly different from those of adult communities (Mothers of 26–35-day-old newborns): ****p < 0.0001, ***p < 0.001, **p < 0.01, *p < 0.05; Wilcoxon rank-sum test adjusted using the Bonferroni correction. Boxplots indicate medians with first and third quartiles (25–75%). Whiskers extend no farther than 1.5*IQR from the hinge.

To measure alpha diversity (the microbial diversity in any single sample), we used both observed richness, the number of distinct OTUs present, and the Shannon Diversity Index, which reflects both richness and evenness. We compared diversity of fecal communities in each age bin to that of the 1-month postpartum mothers, representing an adult microbiota. Alpha diversity of fecal bacterial communities increased with age. Observed bacterial richness was lower than that of adults until 3 years and Shannon diversity until 2 years (Figure 1A). Fungal fecal microbiota diversity of only a few age bins differed from adult diversity; otherwise, there was no clear trend for fungal diversity vs. age, except that it was lower during the first week of life (Figure 1B). Interestingly, the mothers of those newborns also had lower fungal richness than 1-month postpartum mothers. Most samples, especially from after 2 years of age, contained fewer fungal OTUs (less than 60 OTUs in most samples), while between 100 and 300 bacterial OTUs were detected during this time.

### Bacterial and Fungal Communities Across the Age Spectrum

**FIGURE 2:**
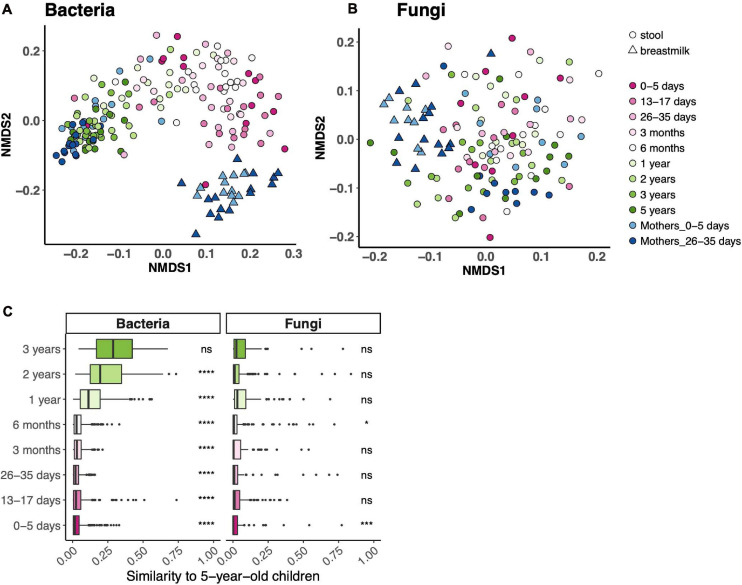
Bacterial and fungal communities across the age spectrum. **(A,B)** Ordinations of community composition based on Bray–Curtis distance for bacterial **(A)** and fungal **(B)** fecal communities. **(C)** Bray–Curtis distance between each age bin and 5 years. ****p < 0.0001, ***p < 0.001, *p < 0.05, Wilcoxon rank-sum test, adjusted using the Bonferroni correction.

Beta diversity (community composition) of fecal bacterial communities differed across the age spectrum and, expectedly, was distinct from breast milk bacterial communities (Figures 2A,B). There was no such trend for fungal fecal communities. While child age contributed to 18% of the variance of fecal bacterial communities (PERMANOVA *p* = 0.001), it accounted for only 2% of variance in fecal fungal communities (PERMANOVA *p* = 0.015). We did not identify one NMDS axis that drove an age gradient for either community. To further assess community composition across the age spectrum, we determined the similarity of communities in each age bin to those at 5 years. Fecal bacterial communities from all age bins, except 3 years, differed from those at 5 years (Figure 2C). Expectedly, fecal fungal communities did not follow this trend.

**FIGURE 3:**
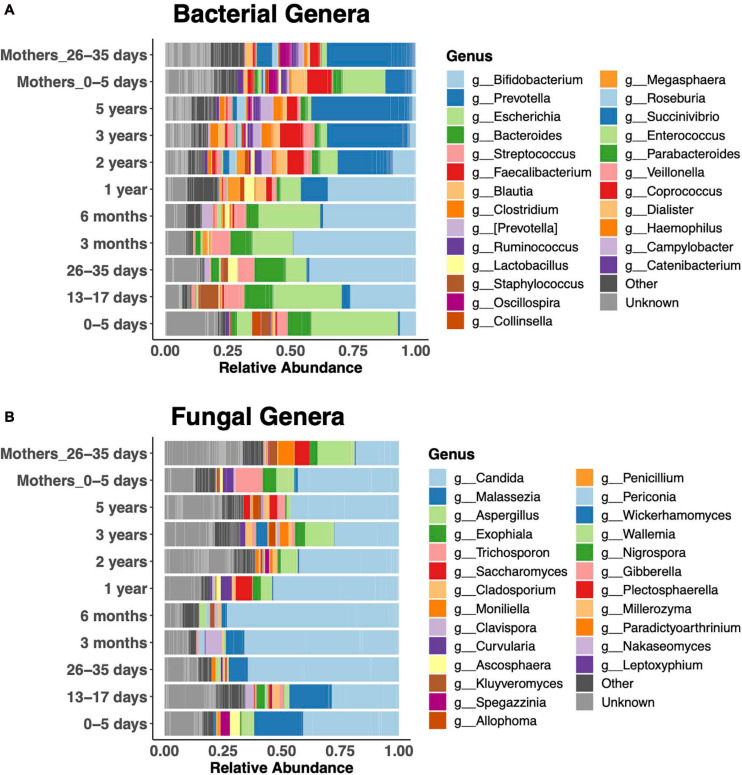
Relative abundance of the 25 most abundant genera. Bacterial **(A)** and fungal **(B)** community composition across the age spectrum.

Relative abundances of the 25 most abundant genera clearly revealed bacterial taxonomic shifts over the age spectrum (Figure 3A). In keeping with the literature on bacteria (Koenig et al., 2011; Stewart et al., 2018), newborns were dominated by *Escherichia coli* in the first week of life, and it remained abundant up to 6 months. Then, *Bifidobacteria* were dominant from 1 month to 1 year. Subsequently, the diversity of genera increased, and *Prevotella* became dominant, similar to the adult microbiota. *Bacteroides* was abundant during the first year, until the increase in *Prevotella*. Children were dominated by either *Bacteroides* or *Prevotella* at any given time (Supplementary Figure 2). *Faecalibacterium* was abundant after the first year. The fecal fungal microbiota was dominated by *Candida* (primarily *Candida albicans* and *Candida tropicalis*) in children of all ages (Figure 3B). *Malassezia* featured prominently in the first 3 months of life, while *Aspergillus* was prominent after the first year.

### Maternal Bacterial Microbiota

**FIGURE 4:**
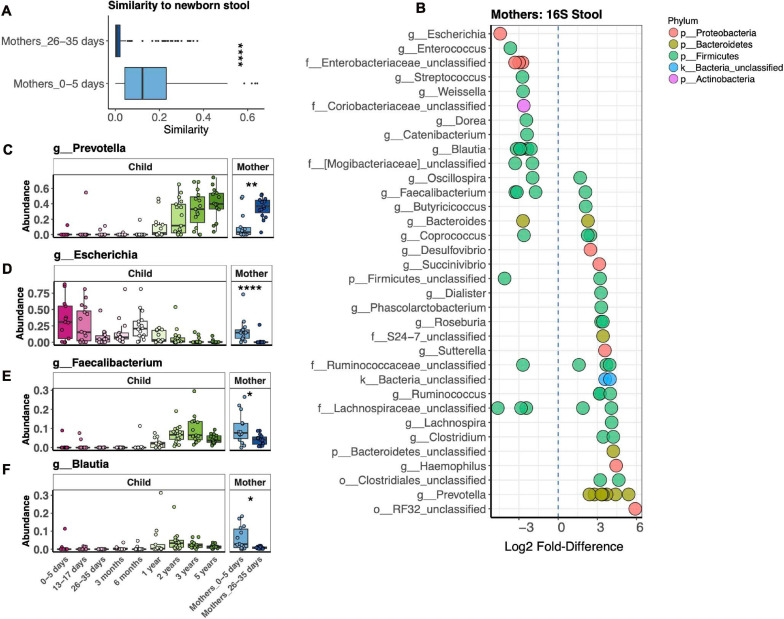
Comparison of fecal bacterial microbiotas of mothers 1 and 4 weeks postpartum and children at 1 week. **(A)** Average pairwise similarity of the microbiotas of mothers vs. those of their children. **(B)** Operational taxonomic units (OTUs) identified by DESeq2 differentially abundant between mothers 1 week vs. 4 weeks postpartum. Negative fold-difference indicates greater abundance 1 week postpartum. **(C–F)** Relative abundance of selected taxa that were differentially abundant between mothers 1 week vs. 4 weeks postpartum. ****p < 0.0001, **p < 0.01, *p < 0.05, Wilcoxon rank-sum test, adjusted using the Bonferroni correction.

Our study revealed dramatic differences in bacterial stool microbiotas of mothers in the first postpartum week vs. 1 month postpartum (Figure 3A). We further explored this difference by measuring the Bray–Curtis distance between mothers’ microbiotas at both time points to their respective children at 1 week, finding that mothers in the first vs. fourth postpartum week harbored a bacterial microbiota significantly more similar to that of newborns (Figure 4A). We identified 68 OTUs differentially abundant between the two mother groups, representing 34 taxa at the genus level or above (Figure 4B). Abundances of some taxa obviously contributed to the similarity of microbiotas of mothers 1 week postpartum and their children at 1 week. Thus, in both mothers and infants from 1 to 4 weeks after birth, *Prevotella* had increasing abundance, while *Escherichia* had decreasing abundance (Figures 4C,D). Other taxa, such as *Faecalibacterium* and *Blautia*, were more abundant in mothers 1 week vs. 4 weeks postpartum, yet were virtually absent in their infants at 1 week (Figures 4E,F). We also found that, at 1 week, newborns shared more bacterial but not fungal OTUs with their mother vs. with unrelated mothers, but this was no longer the case at 1 month (Supplementary Figures 3A,B). OTUs most commonly shared by mother–infant pairs include members of *Escherichia*, *Enterococcus*, and *Streptococcus*, all of which are of higher relative abundance in newborns than in their mothers (Supplementary Figure 3C). Only two fungal OTUs were commonly shared between mother–infant pairs (Supplementary Figure 3D).

### Bacterial and Fungal Communities in Mother’s Breast Milk

**FIGURE 5:**
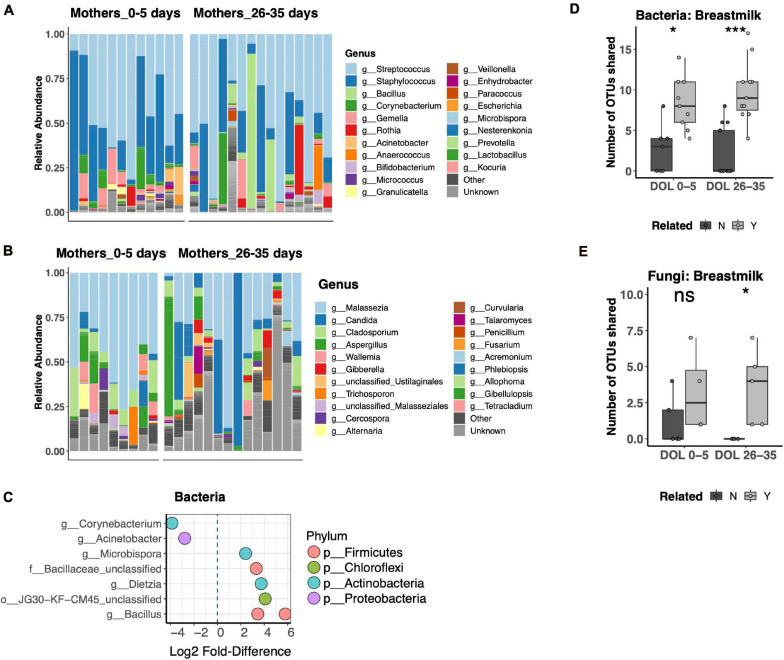
Breast milk bacterial and fungal community composition. **(A,B)** Top 25 most abundant bacterial **(A)** and fungal **(B)** genera across all samples reveal that both communities are dominated by common skin taxa but also contain a diverse repertoire of microbes at lower abundances. **(C)** Differentially abundant taxa between mothers in the first compared to the fourth postpartum week. **(D,E)** Number of shared bacterial **(D)** and fungal **(E)** operational taxonomic units (OTUs) between mother–infant pairs in the first and fourth postpartum weeks. Statistics: ***p < 0.001, *p < 0.05, Wilcoxon rank-sum test, adjusted using the Bonferroni correction.

Breast Milk is a well-recognized modulator of the infant gut microbiota, serving both as a source of colonizing bacteria and bacterial growth substrates. The relationship of breast milk and the fungal microbiota has not been well described. Thus, we compared the bacterial and fungal community composition of both breast milk and fecal microbiotas. Breast milk microbiotas were typically dominated by skin taxa of both bacteria (*Streptococcus*, *Staphylococcus*, or *Corynebacterium*; Figure 5A) and fungi (*Malassezia*, including *Malassezia restricta*, *Malassezia globosa*, *and Malassezia furfur*; Figure 5B). Alpha diversity did not differ between breast milk samples collected in the first or fourth postpartum week for either bacteria or fungi. Only minor differences in community composition were found between the first and fourth postpartum week, including only eight differentially abundant bacterial OTUs (Figure 5C) and no differentially abundant fungal OTUs. For bacterial communities, mother–infant pairs shared significantly more OTUs than unrelated pairs at both postpartum time points (Figure 5D). While fungal data were available for fewer pairs, we found a significant difference between related and unrelated pair sharing 4 weeks postpartum (Figure 5E). The most commonly shared bacterial OTUs belonged to typical skin commensal genera, *Streptococcus* and *Staphylococcus*, which had higher relative abundance in the breast milk than in the newborn stool. Shared OTUs between mother and infant pairs also included gut commensals *Bifidobacterium* and *Bacteroides*, both of which were of higher relative abundance in the newborn’s stool than in his or her mother’s milk (Supplementary Figure 4A). Shared fungal OTUs were much more limited and did not have a clear pattern of differing abundance between mother and child (Supplementary Figure 4B).

## Discussion

Here, we report for the first time the bacterial and fungal microbiota dynamics during early childhood in a rural Ghanaian setting. In conducting a direct comparison between bacterial and fungal gut communities, we demonstrated how the two follow very distinct developmental patterns, having contrasting associations with the mother’s microbiota. We also found a previously unreported and surprising compositional difference in maternal stool microbiotas during the postpartum period.

We found undetectable levels of fungi in a large subset of stool samples from children and mothers in a rural African setting. This finding is consistent with a previous study in Norway, an urban high-resource setting (Schei et al., 2017). We additionally confirmed that the samples with undetectable fungi, including those from the youngest age groups, contained readily amplifiable bacterial genes, providing further evidence that the lack of detectable fungi in these samples reflected their absence or extremely low abundance, rather than sample processing artifacts. However, extraction protocols optimal for bacteria may have failed to lyse fungal cells. Interestingly, most breast milk samples were positive for fungi, especially those from 4 weeks postpartum, while we did not detect fungi in half of the corresponding breastfeeding infants.

As in our study, increasing bacterial (Bokulich et al., 2016; Stewart et al., 2018) and unchanging fungal (Strati et al., 2016; Wampach et al., 2017) richness and diversity during infancy were previously reported, but never together in the same study. However, the timing of increases in bacterial richness and diversity in our study differed from those in other studies in both high- and low-resource regions. In our Ghanaian cohort, richness and diversity remained stable over the first year of life and dramatically increased thereafter. In studies of American and European cohorts, alpha diversity steadily increased from birth to 2 years (Bokulich et al., 2016) or 3 months to 5 years (Stewart et al., 2018), respectively. Numbers of observed OTUs increased steadily from birth to 3 years among Amerindian and Malawian infants (Yatsunenko et al., 2012) and from birth to 2 years in an Indian setting (Chandel et al., 2017). The distinct timing in our study may reflect lifestyles, diet, or an environment unique to this study cohort in Ghana.

The bacterial succession we observed, with sequential dominance by *E. coli* in the first 5 days of life, *Bifidobacterium* and *Bacteroides* in early infancy, and *Prevotella* after the first year, was consistent with previous studies of newborn and adult microbiotas (Koenig et al., 2011; Stewart et al., 2018). The Bacteroidetes phylum in older children and adults is typically dominated by either *Bacteroides* or *Prevotella* (De Filippo et al., 2010; Yatsunenko et al., 2012; Lin et al., 2013). *Prevotella* was dominant in adults in rural Ghana, as demonstrated among the 1-month postpartum women in our study. We found that *Bacteroides* was abundant in the Ghanaian infants prior to 1 year, after which *Prevotella* became dominant. The ability of *Bacteroides* species to utilize human milk oligosaccharides (HMOs) (Marcobal et al., 2011) as well as diet-derived polysaccharides (Flint et al., 2015) as an energy source is thought to contribute to their unique ability to persist in both infant and adult guts. However, *Prevotella* appears to outcompete *Bacteroides* in adults with a fiber-rich diet (Wu et al., 2011). This difference occurs early in life in this Ghanaian population, probably associated with differences in children’s diets at this time.

We found dominance of the gut fungal microbiota of some children by *Candida* sp., as has been found in some (Wampach et al., 2017; Ward et al., 2018) but not other (Strati et al., 2016; Arrieta et al., 2018) studies. We also detected an early increase in the relative abundance of *Malassezia* that was not seen in other studies of newborns (Schei et al., 2017; Ward et al., 2018). Given the high relative abundance of this taxon in breast milk in our study and another (Boix-Amoros et al., 2019) and evidence of its transmission from mother to newborn (Nagata et al., 2012), absence of this taxon in other studies is puzzling but may reflect regional differences in maternal breast milk fungal microbiotas or methodologies.

We were surprised to find substantial differences between the gut bacterial microbiotas of mothers 1 week vs. 4 weeks postpartum. These dramatic differences were not due to cross-contamination between maternal and infant samples, as mothers in both groups were sampled using the same protocol, and samples from both groups were processed simultaneously. Given the cross-sectional study design, we cannot definitively conclude that the microbiota changed in these women over this time. However, both the 3-month recruitment window and the greater microbiota similarity of mothers and their infants 1 week postpartum imply that such a change is the most likely interpretation of the data. Studies of the stool microbiota during pregnancy have yielded mixed results, with some studies showing no change in community composition during pregnancy (Bisanz et al., 2015; DiGiulio et al., 2015) and others showing substantial shifts (Koren et al., 2012; Ferrocino et al., 2018). The latter two studies showed an increase in Proteobacteria during pregnancy, which is consistent dominance of Proteobacteria that we observed 1 week postpartum. However, our finding of a low relative abundance of *Prevotella* 1 week postpartum is novel and was one of the greatest differences we observed in the postpartum period. We also observed a high relative abundance of *Faecalibacterium* 1 week postpartum, associated with decreased inflammation and protection from inflammatory bowel diseases (Sokol et al., 2008), and *Blautia*, negatively associated with type 1 diabetes (Murri et al., 2013) and gestational diabetes mellitus (GDM) without prescribed dietary intervention (Ferrocino et al., 2018). Together, our findings suggest that differences in maternal microbiota around the time of birth reflect both increases in taxa common to newborns (*Escherichia*) and decreases in taxa absent in newborns (*Prevotella*), as well as increases in abundance of taxa not abundant in infants but with potential health benefits to the mother (*Faecaibacterim* and *Blautia*).

We did not identify fungal taxa that were differentially abundant in the gut microbiota 1 week vs. 4 weeks postpartum. We also did not identify many commonly shared fungal OTUs between pairs. In contrast to the bacterial gut microbiota, the fungal gut microbiota of mothers does not appear to be substantially affected by postpartum time and does not appear to strongly influence colonization of the infant’s gut using the methods we applied.

We found that common skin taxa dominated breast milk bacterial (*Streptococcus*, *Staphylococcus*) and fungal (*Malassezia*) communities, as previously shown (Murphy et al., 2017; Williams et al., 2017; Boix-Amoros et al., 2019; Moossavi et al., 2019). When dominant in a mother’s breast milk, OTUs of these taxa tended to be present at lower relative abundance in her infant’s gut microbiota, consistent with the breast milk being a source for these organisms colonizing the infant gut. However, it is important to note that detection of their DNA in breast milk and feces does not demonstrate that these OTUs colonize, or even survive, these two environments. Further investigation is required to determine if these skin taxa have any function in breast milk or the gut. OTUs of common gut taxa (*Bifidobacterium*, *Bacteroides*, *Lactobacillus*, and *Rothia*) were also frequently shared between a mother’s breast milk and her infant’s gut microbiota, as previously reported (Murphy et al., 2017). However, the gut commensals tended to have higher relative abundance in the gut than the breast milk, suggesting that the infant’s (or mother’s) gut was a source for these organisms colonizing the breast milk. Overall, our study identifies strong and novel associations between the microbiotas of breast milk and the infant gut, particularly in the case of bacteria vs. fungi.

There are limitations in the study worth noting. Amplicon Sequence Variants (ASVs) are becoming an increasingly popular approach as an alternative to the OTU clustering performed in our study. However, we submit that ASV analysis would only have a minor impact on our conclusions based on a recent study demonstrating that both ASV and OTU approaches reveal biologically meaningful and comparable results (Glassman and Martiny, 2018). With this, others have cautioned against abandoning OTU approaches for sequencing applications targeting a single variable region (Johnson et al., 2019). Independent of sequencing approach and given the cross-sectional design, this study cannot definitively prove that differences among age groups represent microbiota changes over time, although such changes are the most compelling interpretation of the results. Sample size and a lack of data on infant and mother health and on mode of delivery limited the analysis to effects of age but did not include other host factors that also may have influenced the microbiotas.

## Conclusion

The goal of this study was to assess, for the first time, the bacterial and fungal gut microbiota composition of children in Ghana to help identify microbial signatures relevant to the health of women and their children. In doing so, we not only identified bacterial and fungal community dynamics shared with other study cohorts but also identified characteristics not previously reported. Unique to the Ghanaian cohort, alpha diversity was low and stable during the first year of life, and *Prevotella* became the dominant bacterial genus between the first and second year. Also, the mother’s bacterial microbiota differed drastically between 1 and 4 weeks postpartum. While mothers are a major source of bacteria colonizing newborns, the sources of colonizing fungi are less apparent.

## Future Directions

This small cross-sectional study broadly assessed ecology mother–newborn bacterial and fungal microbiotas, together with the microbiota in children over the first 5 years in rural Ghana. Crucial insights from this study inform future approaches to identify microbial signatures relevant to the health of women and their children. Based on our findings, longitudinal studies will address the following questions:

1. What demographic or environmental factors explain differences in maternal microbiotas after childbirth? And are these changes associated with maternal health or disease?
2. Are there host microbiota (bacterial and fungal) signatures associated with increased risk for, or protection from, severe disease, especially in the newborn period?
3. Are commensal fungi identified in mother–infant pairs transient or do they represent a stable community of commensal microbes coinhabiting the gut with bacteria?

These studies will inform novel interventions targeting the microbiota to improve the health of mothers and their children.

## Data Availability Statement

The datasets presented in this study can be found in online repositories. The names of the repository/repositories and accession number(s) can be found below: https://www.ncbi.nlm.nih.gov/, bioproject/PRJNA658595. All codes and data used to generate the results presented in this manuscript are publicly accessible on GitHub using the following URL: https://github.com/nelly-amenyogbe/Bacterial_Fungal_Communities_Rural_Ghana.

## Ethics Statement

The studies involving human participants were reviewed and approved by All work in this study was in accordance to ethical guidelines at the University of British Columbia and done under the approved ethics protocol number H11-01423. Separate ethical approval was obtained from the Kintampo Health Research Centre Institutional Ethics Committee (KHRCIEC_2016-14). For participants under the age of 18, Written informed consent to participate in this study was provided by the participants’ legal guardian/next of kin.

## Author Contributions

SO-A, DA-G, NA, TK, KA, YE, and PP contributed to the study design. DA-G, YE, SK, and DD facilitated participant recruitment and sample collection. NA and WM generated the amplicon sequencing data. NA performed the statistical analyses and prepared the manuscript. WM and SO-A substantially edited the manuscript. All authors contributed to editing the manuscript.

## Conflict of Interest

The authors declare that the research was conducted in the absence of any commercial or financial relationships that could be construed as a potential conflict of interest.

## Footnotes

1 https://www.mothur.org/wiki/MiSeq_SOP

## References

[B1] Arrieta M. C. Arevalo A. Stiemsma L. Dimitriu P. Chico M. E. Loor S. (2018). Associations between infant fungal and bacterial dysbiosis and childhood atopic wheeze in a nonindustrialized setting. *J. Allergy Clin. Immunol.* 142 424–434.e10. 29241587 10.1016/j.jaci.2017.08.041 PMC6075469

[B2] Arrieta M. C. Stiemsma L. T. Amenyogbe N. Brown E. M. Finlay B. (2014). The intestinal microbiome in early life: health and disease. *Front. Immunol.* 5:427. 25250028 10.3389/fimmu.2014.00427 PMC4155789

[B3] Backhed F. Roswall J. Peng Y. Feng Q. Jia H. Kovatcheva-Datchary P. (2015). Dynamics and stabilization of the human gut microbiome during the first year of life. *Cell Host Microbe* 17 690–703. 25974306 10.1016/j.chom.2015.04.004

[B4] Bisanz J. E. Enos M. K. PrayGod G. Seney S. Macklaim J. M. Chilton S. (2015). Microbiota at multiple body sites during pregnancy in a rural tanzanian population and effects of moringa-supplemented probiotic yogurt. *Appl. Environ. Microbiol.* 81 4965–4975. 10.1128/aem.00780-15 25979893 PMC4495201

[B5] Boix-Amoros A. Puente-Sanchez F. du Toit E. Linderborg K. M. Zhang Y. Yang B. (2019). Mycobiome profiles in breast milk from healthy women depend on mode of delivery, geographic location and interaction with bacteria. *Appl. Environ. Microbiol.* 85 e2994–e2918. 10.1128/AEM.02994-18 PMC6495746 30824446

[B6] Bokulich N. A. Chung J. Battaglia T. Henderson N. Jay M. Li H. (2016). Antibiotics, birth mode, and diet shape microbiome maturation during early life. *Sci. Transl. Med.* 8:343ra82. 10.1126/scitranslmed.aad7121 27306664 PMC5308924

[B7] Cabrera-Rubio R. Collado M. C. Laitinen K. Salminen S. Isolauri E. Mira A. (2012). The human milk microbiome changes over lactation and is shaped by maternal weight and mode of delivery. *Am. J. Clin. Nutr.* 96 544–551. 10.3945/ajcn.112.037382 22836031

[B8] Chandel D. S. Perez-Munoz M. E. Yu F. Boissy R. Satpathy R. Misra P. R. (2017). Changes in the gut microbiota after early administration of oral synbiotics to young infants in India. *J. Pediatr. Gastroenterol. Nutr.* 65 218–224. 10.1097/mpg.0000000000001522 28121648 PMC5524612

[B9] Charlop-Powers Z. Brady S. F. (2015). phylogeo: an R package for geographic analysis and visualization of microbiome data. *Bioinformatics* 31 2909–2911. 10.1093/bioinformatics/btv269 25913208 PMC4547612

[B10] De Filippo C. Cavalieri D. Di Paola M. Ramazzotti M. Poullet J. B. Massart S. (2010). Impact of diet in shaping gut microbiota revealed by a comparative study in children from Europe and rural Africa. *Proc. Natl. Acad. Sci. U.S.A.* 107 14691–14696. 10.1073/pnas.1005963107 20679230 PMC2930426

[B11] DeSantis T. Z. Hugenholtz P. Larsen N. Rojas M. Brodie E. L. Keller K. (2006). Greengenes, a chimera-checked 16S rRNA gene database and workbench compatible with ARB. *Appl. Environ. Microbiol.* 72 5069–5072. 10.1128/aem.03006-05 16820507 PMC1489311

[B12] DiGiulio D. B. Callahan B. J. McMurdie P. J. Costello E. K. Lyell D. J. Robaczewska A. (2015). Temporal and spatial variation of the human microbiota during pregnancy. *Proc. Natl. Acad. Sci. U.S.A.* 112 11060–11065. 26283357 10.1073/pnas.1502875112 PMC4568272

[B13] Ferrocino I. Ponzo V. Gambino R. Zarovska A. Leone F. Monzeglio C. (2018). Changes in the gut microbiota composition during pregnancy in patients with gestational diabetes mellitus (GDM). *Sci. Rep.* 8:12216. 30111822 10.1038/s41598-018-30735-9 PMC6093919

[B14] Flint H. J. Duncan S. H. Scott K. P. Louis P. (2015). Links between diet, gut microbiota composition and gut metabolism. *Proc. Nutr. Soc.* 74 13–22. 10.1017/s0029665114001463 25268552

[B15] Glassman S. I. Martiny J. B. H. (2018). Broadscale ecological patterns are robust to use of exact sequence variants versus operational taxonomic units. *mSphere* 3 e148–e118. 10.1128/mSphere.00148-18 PMC6052340 30021874

[B16] Gweon H. S. Oliver A. Taylor J. Booth T. Gibbs M. Read D. S. (2015). PIPITS: an automated pipeline for analyses of fungal internal transcribed spacer sequences from the Illumina sequencing platform. *Methods Ecol. Evol.* 6 973–980. 10.1111/2041-210x.12399 27570615 PMC4981123

[B17] Heisel T. Nyaribo L. Sadowsky M. J. Gale C. A. (2019). Breastmilk and NICU surfaces are potential sources of fungi for infant mycobiomes. *Fungal Genet. Biol.* 128 29–35. 10.1016/j.fgb.2019.03.008 30905830 PMC6555646

[B18] Jiang T. T. Shao T. Y. Ang W. X. G. Kinder J. M. Turner L. H. Pham G. (2017). Commensal fungi recapitulate the protective benefits of intestinal bacteria. *Cell Host Microbe* 22 809–816.e4. 29174402 10.1016/j.chom.2017.10.013 PMC5730478

[B19] Jimenez E. Delgado S. Maldonado A. Arroyo R. Albujar M. Garcia N. (2008). Staphylococcus epidermidis: a differential trait of the fecal microbiota of breast-fed infants. *BMC Microbiol.* 8:143. 10.1186/1471-2180-8-143 18783615 PMC2551609

[B20] Johnson J. S. Spakowicz D. J. Hong B. Y. Petersen L. M. Demkowicz P. Chen L. (2019). Evaluation of 16S rRNA gene sequencing for species and strain-level microbiome analysis. *Nat. Commun.* 10:5029. 31695033 10.1038/s41467-019-13036-1 PMC6834636

[B21] Jost T. Lacroix C. Braegger C. Chassard C. (2013). Assessment of bacterial diversity in breast milk using culture-dependent and culture-independent approaches. *Br. J. Nutr.* 110 1253–1262. 10.1017/s0007114513000597 23507238

[B22] Koenig J. E. Spor A. Scalfone N. Fricker A. D. Stombaugh J. Knight R. (2011). Succession of microbial consortia in the developing infant gut microbiome. *Proc. Natl. Acad. Sci. U.S.A.* 108(Suppl. 1) 4578–4585. 10.1073/pnas.1000081107 20668239 PMC3063592

[B23] Koljalg U. Larsson K. H. Abarenkov K. Nilsson R. H. Alexander I. J. Eberhardt U. (2005). UNITE: a database providing web-based methods for the molecular identification of ectomycorrhizal fungi. *New Phytol.* 166 1063–1068. 10.1111/j.1469-8137.2005.01376.x 15869663

[B24] Koren O. Goodrich J. K. Cullender T. C. Spor A. Laitinen K. Backhed H. K. (2012). Host remodeling of the gut microbiome and metabolic changes during pregnancy. *Cell* 150 470–480. 10.1016/j.cell.2012.07.008 22863002 PMC3505857

[B25] Kozich J. J. Westcott S. L. Baxter N. T. Highlander S. K. Schloss P. D. (2013). Development of a dual-index sequencing strategy and curation pipeline for analyzing amplicon sequence data on the MiSeq Illumina sequencing platform. *Appl. Environ. Microbiol.* 79 5112–5120. 10.1128/aem.01043-13 23793624 PMC3753973

[B26] Lin A. Bik E. M. Costello E. K. Dethlefsen L. Haque R. Relman D. A. (2013). Distinct distal gut microbiome diversity and composition in healthy children from Bangladesh and the United States. *PLoS One* 8:e53838. 10.1371/journal.pone.0053838 23349750 PMC3551965

[B27] Love M. I. Huber W. Anders S. (2014). Moderated estimation of fold change and dispersion for RNA-seq data with DESeq2. *Genome Biol.* 15 550. 25516281 10.1186/s13059-014-0550-8 PMC4302049

[B28] Marcobal A. Barboza M. Sonnenburg E. D. Pudlo N. Martens E. C. Desai P. (2011). *Bacteroides* in the infant gut consume milk oligosaccharides via mucus-utilization pathways. *Cell Host Microbe* 10 507–514. 10.1016/j.chom.2011.10.007 22036470 PMC3227561

[B29] Martin V. Maldonado-Barragan A. Moles L. Rodriguez-Banos M. Campo R. D. Fernandez L. (2012). Sharing of bacterial strains between breast milk and infant feces. *J. Hum. Lact.* 28 36–44. 10.1177/0890334411424729 22267318

[B30] Moossavi S. Fehr K. Derakhshani H. Sbihi H. Robertson B. Bode L. (2020). Human milk fungi: environmental determinants and inter-kingdom associations with milk bacteria in the CHILD Cohort Study. *BMC Microbiol.* 20:146. 10.1186/s12866-020-01829-0 32503420 PMC7275434

[B31] Moossavi S. Sepehri S. Robertson B. Bode L. Goruk S. Field C. J. (2019). Composition and variation of the human milk microbiota are influenced by maternal and early-life factors. *Cell Host Microbe* 25 324.e–335.e. 30763539 10.1016/j.chom.2019.01.011

[B32] Murphy K. Curley D. O’Callaghan T. F. O’Shea C. A. Dempsey E. M. O’Toole P. W. (2017). The composition of human milk and infant faecal microbiota over the first three months of life: a pilot study. *Sci. Rep.* 7: 40597. 10.1038/srep40597 PMC5240090 28094284

[B33] Murri M. Leiva I. Gomez-Zumaquero J. M. Tinahones F. J. Cardona F. Soriguer F. (2013). Gut microbiota in children with type 1 diabetes differs from that in healthy children: a case-control study. *BMC Med.* 11:46. 10.1186/1741-7015-11-46 23433344 PMC3621820

[B34] Nagata R. Nagano H. Ogishima D. Nakamura Y. Hiruma M. Sugita T. (2012). Transmission of the major skin microbiota, Malassezia, from mother to neonate. *Pediatr. Int.* 54 350–355. 10.1111/j.1442-200x.2012.03563.x 22300401

[B35] Oksanen J. Blanchet G. Friendly M. Kindt R. Legendre P. McGlinn D. (2017). *vegan: Community Ecology Package.* Available online at: https://CRAN.R-project.org/package=vegan.

[B36] Palmer C. Bik E. M. DiGiulio D. B. Relman D. A. Brown P. O. (2007). Development of the human infant intestinal microbiota. *PLoS Biol.* 5:e177. 10.1371/journal.pbio.0050177 17594176 PMC1896187

[B37] Schei K. Avershina E. Oien T. Rudi K. Follestad T. Salamati S. (2017). Early gut mycobiota and mother-offspring transfer. *Microbiome* 5:107. 28837002 10.1186/s40168-017-0319-x PMC5571498

[B38] Schloss P. D. Westcott S. L. Ryabin T. Hall J. R. Hartmann M. Hollister E. B. (2009). Introducing mothur: open-source, platform-independent, community-supported software for describing and comparing microbial communities. *Appl. Environ. Microbiol.* 75 7537–7541. 10.1128/aem.01541-09 19801464 PMC2786419

[B39] Shao T. Y. Ang W. X. G. Jiang T. T. Huang F. S. Andersen H. Kinder J. M. (2019). Commensal candida albicans positively calibrates systemic th17 immunological responses. *Cell Host Microbe* 25 404–417.e6. 30870622 10.1016/j.chom.2019.02.004 PMC6419754

[B40] Sokol H. Pigneur B. Watterlot L. Lakhdari O. Bermudez-Humaran L. G. Gratadoux J. J. (2008). Faecalibacterium prausnitzii is an anti-inflammatory commensal bacterium identified by gut microbiota analysis of Crohn disease patients. *Proc. Natl. Acad. Sci. U.S.A.* 105 16731–16736. 10.1073/pnas.0804812105 18936492 PMC2575488

[B41] Solis G. de Los Reyes-Gavilan C. G. Fernandez N. Margolles A. Gueimonde M. (2010). Establishment and development of lactic acid bacteria and bifidobacteria microbiota in breast-milk and the infant gut. *Anaerobe* 16 307–310. 10.1016/j.anaerobe.2010.02.004 20176122

[B42] Stewart C. J. Ajami N. J. O’Brien J. L. Hutchinson D. S. Smith D. P. Wong M. C. (2018). Temporal development of the gut microbiome in early childhood from the TEDDY study. *Nature* 562 583–588. 30356187 10.1038/s41586-018-0617-x PMC6415775

[B43] Strati F. Di Paola M. Stefanini I. Albanese D. Rizzetto L. Lionetti P. (2016). Age and gender affect the composition of fungal population of the human gastrointestinal tract. *Front. Microbiol.* 7:1227. 10.3389/fmicb.2016.01227 27536299 PMC4971113

[B44] Subramanian S. Huq S. Yatsunenko T. Haque R. Mahfuz M. Alam M. A. (2014). Persistent gut microbiota immaturity in malnourished Bangladeshi children. *Nature* 510 417–421. 10.1038/nature13421 24896187 PMC4189846

[B45] Wampach L. Heintz-Buschart A. Hogan A. Muller E. E. L. Narayanasamy S. Laczny C. C. (2017). Colonization and succession within the human gut microbiome by archaea, bacteria, and microeukaryotes during the first year of life. *Front. Microbiol.* 8:738. 10.3389/fmicb.2017.00738 28512451 PMC5411419

[B46] Ward T. L. Dominguez-Bello M. G. Heisel T. Al-Ghalith G. Knights D. Gale C. A. (2018). Development of the human mycobiome over the first month of life and across body sites. *mSystems* 3 e00140–17. 29546248 10.1128/mSystems.00140-17 PMC5840654

[B47] Williams J. E. Carrothers J. M. Lackey K. A. Beatty N. F. York M. A. Brooker S. L. (2017). Human milk microbial community structure is relatively stable and related to variations in macronutrient and micronutrient intakes in healthy lactating women. *J. Nutr.* 147 1739–1748. 28724659 10.3945/jn.117.248864 PMC5572491

[B48] Wu G. D. Chen J. Hoffmann C. Bittinger K. Chen Y. Y. Keilbaugh S. A. (2011). Linking long-term dietary patterns with gut microbial enterotypes. *Science* 334 105–108. 10.1126/science.1208344 21885731 PMC3368382

[B49] Yatsunenko T. Rey F. E. Manary M. J. Trehan I. Dominguez-Bello M. G. Contreras M. (2012). Human gut microbiome viewed across age and geography. *Nature* 486 222–227. 10.1038/nature11053 22699611 PMC3376388

