# Supplementary material for: Bacterial and Fungal Gut Community Dynamics Over the First 5 Years of Life in Predominantly Rural Communities in Ghana
Source: Front Microbiol. 2021 Jul 6;12:664407. doi: 10.3389/fmicb.2021.664407 (PMC8290483; doi:10.3389/fmicb.2021.664407)
Supplement: Supplementary file 1 [file Data_Sheet_1.docx]

Supplementary Material

# Supplementary Table

**Sample Summary**

|  | n | Sex: n (%) | |
| --- | --- | --- | --- |
|  |  | Female | Male |
| 0-5 days | 15 | 9 (60.0) | 6 (40.0) |
| 13-17 days | 16 | 8 (50.0) | 8 (50.0) |
| 26-35 days | 15 | 4 (26.7) | 11 (73.3) |
| 3 months | 16 | 5 (31.2) | 11 (68.8) |
| 6 months | 14 | 7 (50.0) | 7 (50.0) |
| 1 year | 13 | 8 (61.5) | 5 (38.5) |
| 2 years | 17 | 9 (52.9) | 8 (47.1) |
| 3 years | 16 | 7 (43.8) | 9 (56.2) |
| 5 years | 15 | 8 (53.3) | 7 (46.7) |
| Mothers_0-5 days | 15 | 15 | 0 (0.0) |
| Mothers_26-35 days | 15 | 15 | 0 (0.0) |

## Supplementary Figures

**
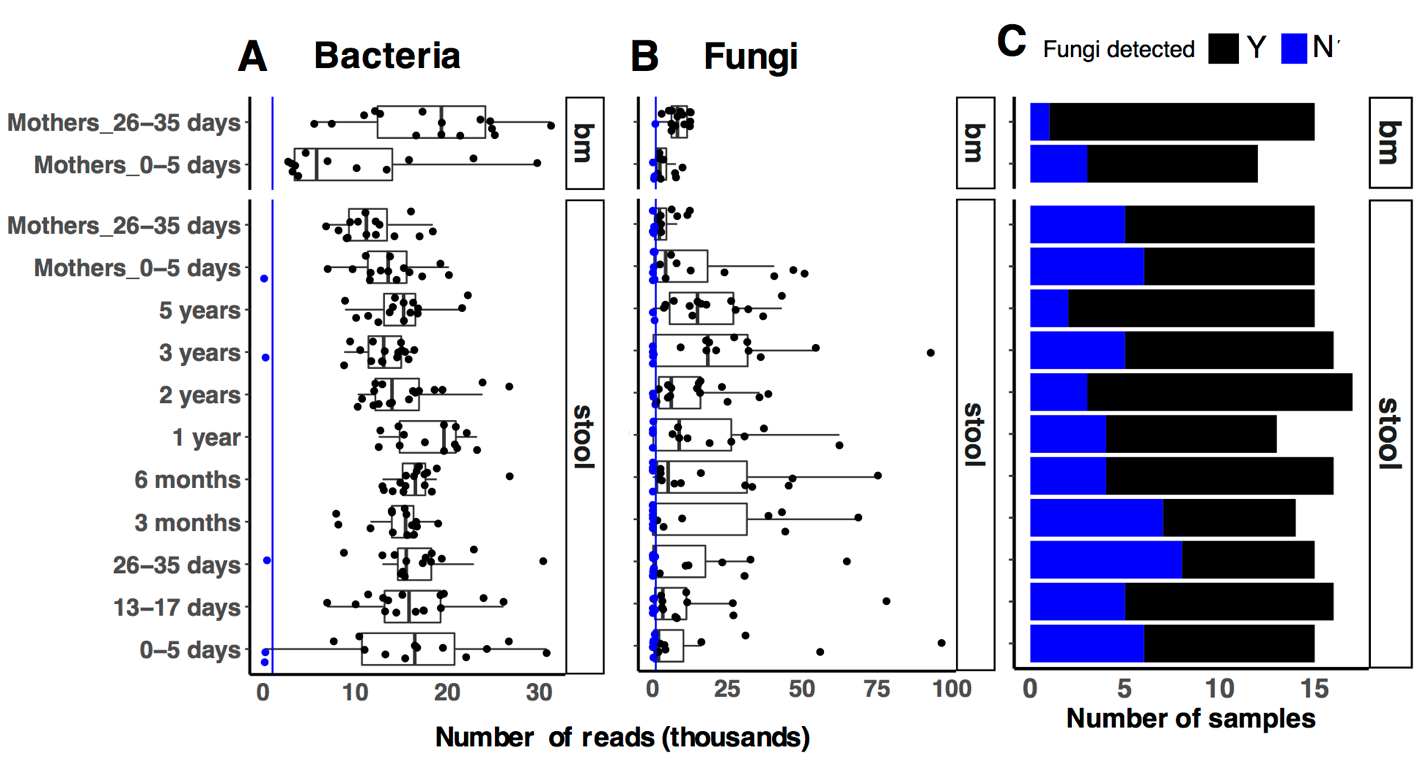
Supplementary Figure 1. Amplicon sequencing coverage for bacterial and fungal communities in each age stratification.** A. Amplicon sequencing allowed for bacterial profiling of nearly all samples at a sequencing depth over 10,000 for most samples. B. For fungal profiling, several samples did not produce sufficient ITS2 sequencing reads for downstream analysis. C. Samples with no detectable fungi spanned the age spectrum. Abbreviations: bm, breast milk.


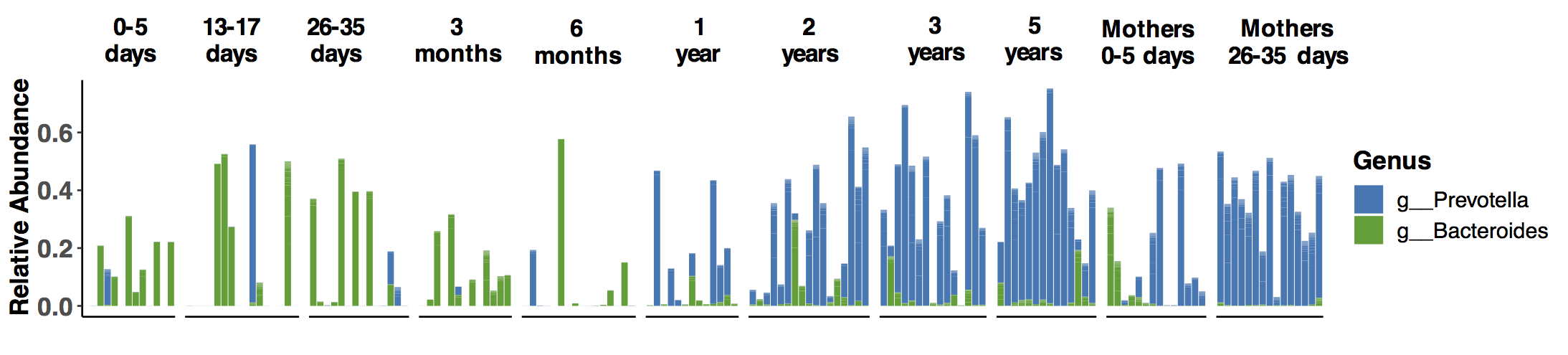


**Supplementary Figure 2. Relative Abundance of *Bacteroides*  and *Prevotella* genera in individual fecal samples**

**
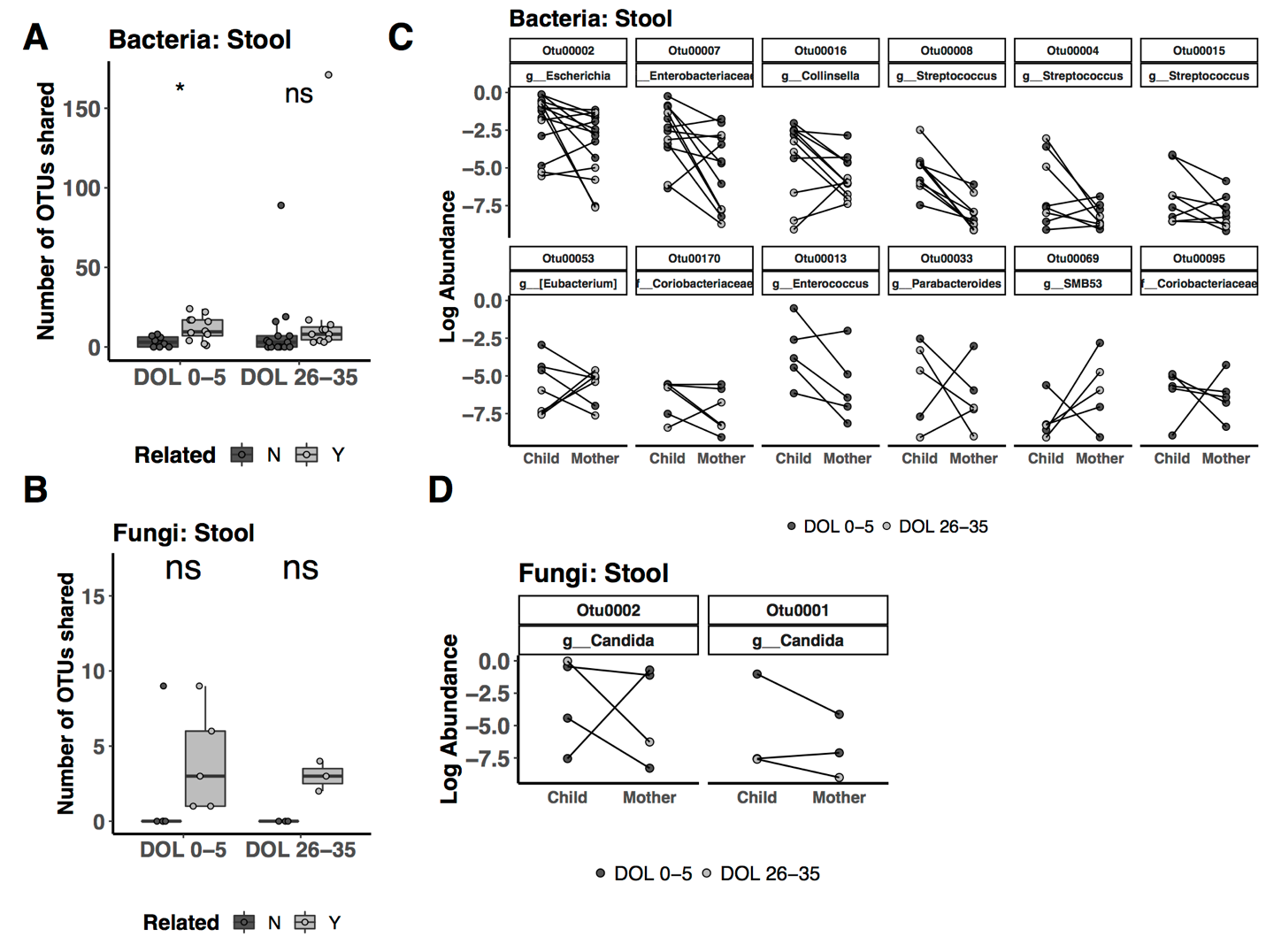
Supplementary Figure 3. Shared OTUs between mother’s and newborn’s stool.** A,B. Number of shared bacterial (A) and fungal (B) OTUs between mother-infant pairs in the first and fourth post-partum weeks. Statistics: G. * p < 0.05, Wilcoxon Rank Sum test, adjusted using the Bonferroni correction. C,D. Log Relative abundance of bacterial (C) and fungal (D) OTUs most commonly shared between stools of a mother and her newborn.

**
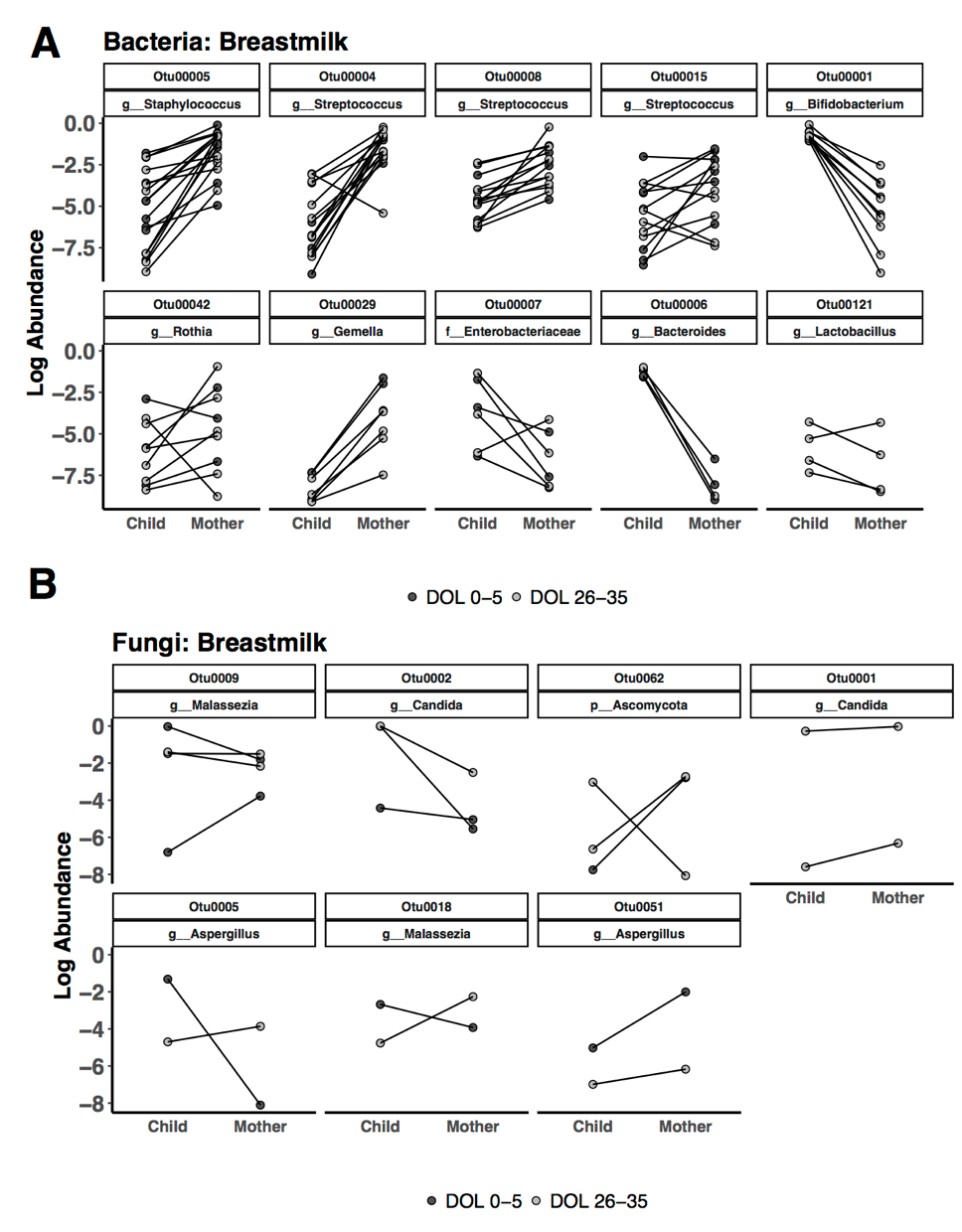
Supplementary Figure 4. Log Relative abundance of bacterial and fungal taxa shared by mother-infant pairs.** (A) Bacterial and (B) fungal OTUs most commonly shared between a mother’s breastmilk and her newborn’s stool.
